# Does inhabitant’s fertility intention respond to housing status in the urban built environment: evidence from China

**DOI:** 10.3389/fpubh.2025.1328329

**Published:** 2025-03-19

**Authors:** Xuefang Zhuang, Qingyin Li, Tian Su, Siying Zhang, Rong Wu, Tianxiang Zheng

**Affiliations:** ^1^School of Architecture and Urban Planning, Guangdong University of Technology, Guangzhou, China; ^2^Shenzhen Tourism College, Jinan University, Shenzhen, China; ^3^Department of E-Commerce, Jinan University, Shenzhen, China

**Keywords:** fertility intention, housing status, housing mortgage, regional diversity, mental health

## Abstract

**Background:**

The total fertility rate in China has been dropping in recent years, and this continuing trend has led to a series of problems. China has experienced periods of urbanization and housing reforms, leading to a significant boom in the real estate market. Housing status appears to be an essential factor influencing the fertility-related decisions of residents in China.

**Methods:**

We use cross-sectional data from a nationally representative large-scale tracking survey targeting the labor force in China. The research sample for this study comprised data from Chinese adults between the ages of 20 and 45. We highlight the importance of housing mortgages on fertility intention based on the Poisson regression model.

**Results:**

Data was collected from a total of 7,512 inhabitants. The results show the following: 1) Housing status is closely related to fertility intention, while housing mortgage and water contamination are negatively affected. 2) The urban built environment, social environment, and individual characteristics affecting fertility intention mainly occur in urbanization rate, green coverage rate in urban built-up areas (GCR), life satisfaction, mental health, age, gender, marital status, political status, and education status. Urbanization rate, GCR, life satisfaction, and mental health positively influence resident’s fertility intention, whereas women and single show lower fertility intention. 3) In particular, there are significant regional differences in the mechanism of fertility intention. Such intention in the eastern and central regions is primarily related to housing mortgages, urban built environment, the degree of contamination in the habitat, and socioeconomic factors, while the intention in the northeastern region is related to soil contamination. Fertility intention in the western and northeastern region are strongly related to mental health.

## 1 Introduction

The results published by the National Bureau of Statistics of China in 2022 marked the first instance of negative population development in 61 years, with a birth rate of only 6.77‰. This suggests that the anticipated effect of the government’s successive implementation of the two-child and three-child policies on fertility stimulation has not been as substantial. As fears of overpopulation in China grew in the 1970s, the government implemented the one-child policy to control its rate of population growth after the Reform and Opening Up in 1979 (1–3). It led to a rapid change in the country’s pattern of population growth. In recent years, however, the overall fertility rate in China has been declining, and a continuation of this trend is expected to lead to a series of problems, including gender imbalance (4), accelerated population aging (5), and labor shortage (6). To mitigate such a decline, the Chinese government transformed its fertility policy in 2011 and began encouraging childbearing instead of discouraging it. Policies, including the “selective two-child” policy in 2013 and the “universal two-child” policy in 2016, were promulgated to maintain the long-term population balance and alleviate the pressure brought about by an aging population (7, 8). However, these adjustments have not been able to reverse the trend of declining fertility rates (7). According to the Seventh Census in 2020, the fertility rate in China has fallen to 1.3. This number is much lower than the replacement fertility rate of 2.11 required to maintain the population. In light of the census results, China adopted the “three-child” fertility policy in 2021 to raise the national fertility rate (9). Unlike the natural process of declining fertility in developed countries, China’s low fertility rate is thus a product of both the government’s fertility policies and the country’s economic development. This situation is exacerbated by its extent of economic development, which remains below that of many developed countries, and an imperfect social security system for the older adult (10).

Many studies have focused on individual characteristics that influence fertility intention, including time, energy, and financial costs. Some scholars have examined the relationships between women’s education and employment/occupation and their fertility intention (8, 11–13). A positive association has been observed in general between women’s level of education and their fertility intention. However, Alcaraz et al. found that the desire for education and childbearing were inversely related to each other (14). Employment-related factors have different effects on the fertility intention of people in different areas. For instance, Han et al. observed that employed women were less likely to have fertility intention than unemployed women (15). Similar results were reported by Begall, who revealed that higher “attachment” to the labor force, indicated by the number of years in paid employment, among women without children was associated with lower fertility intention (16). Budig demonstrated that both part-time and full-time jobs in the United States reduced the likelihood of fertility intention among women (17). Researchers have also attempted to show that women employed in jobs with higher potential earnings were more likely to postpone childbearing but had higher intention to give birth than those not (18). Others have suggested that the incompatibility between paid labor and household chores is the main reason women control unintended births (19).

Most prevalent research also shows that economic factors related to housing, such as house prices and home ownership, affect people’s fertility intention (20–22). Marriage is considered to be necessary to have children in Chinese society, and property ownership before marriage is considered an important economic foundation for the new family. Family income has long been shown to increase people’s fertility intention (23). A house is considered to be a place for settling down and raising children in traditional Chinese culture. The concept of “buying a house before getting married” has become a social custom in China over recent decades (24, 25). Following the Reform and Opening Up, as well as the orientation of the real estate economy, China’s real estate entered a long boom due to the influx of people into large cities and the promotion of market speculation. This led to a rapid increase in housing prices and housing expenses, especially mortgage payments in many cities, which have become a major burden for young people (26). Rising prices of housing increase household income for homeowners in the short term (20). However, they also increase renters’ cost of living, such that the impacts of fluctuations in housing prices on the fertility intention of these two groups are significantly different (22, 27). Researchers in many countries have shown that as housing prices rise, the fertility intention of homeowners rises but that of renters falls. High house prices affect the rates of marriage and, indirectly, the rate of first births, but they also reduce the residents’ willingness to have children (28). Their negative effect on people’s willingness to have one child is much greater than on their intention to have two children (29). Fluctuations in house prices have a smaller influence on the fertility intention of people in higher-income groups. The Chinese government has encouraged childbirth and regulated increases in housing prices in recent years, but these measures have failed to promote fertility. This shows that the fertility intention of people is not influenced only by government policies and the economy today.

Moreover, urbanization is a crucial influence on fertility intention in the urban environment. Several studies in Europe and China have identified a declining trend in fertility during the urbanization process. In particular, the negative influence of urbanization on fertility has gradually outweighed the lagging effects of fertility policy in China (30–33). There are also regional differences in the effect of urbanization on fertility, with fertility disparities being exacerbated by the combined effects of positive income, negative substitution effects, and congestion diseconomies when populations migrate from areas with low urbanization rates to areas with high urbanization rates (33, 34). However, these changes are phased as urbanization-induced population decline and reduce urban congestion, which increases the fertility rate (31). In addition, childcare policy, land use, road network density, and population density in cities have an important effect on fertility rates (33, 35, 36). Otherwise, environmental quality is another significant factor affecting population fertility in the process of urbanization. Numerous studies have demonstrated the negative impingement of air pollution on fertility intention and birth quality, and a study in China has discovered that PM2.5 has a significant inhibiting effect on the second birth intention of the floating population (37, 38). An increase in environmental quality perception can significantly promote people’s fertility intention, which is more prominent for people with poor socioeconomic conditions (32, 38). Furthermore, urban green spaces can mitigate the negative effects of environmental pollution on fertility, and numerous studies indicate that increased exposure to green spaces can enhance fertility quality and intention (39–43).

**Figure 1:**
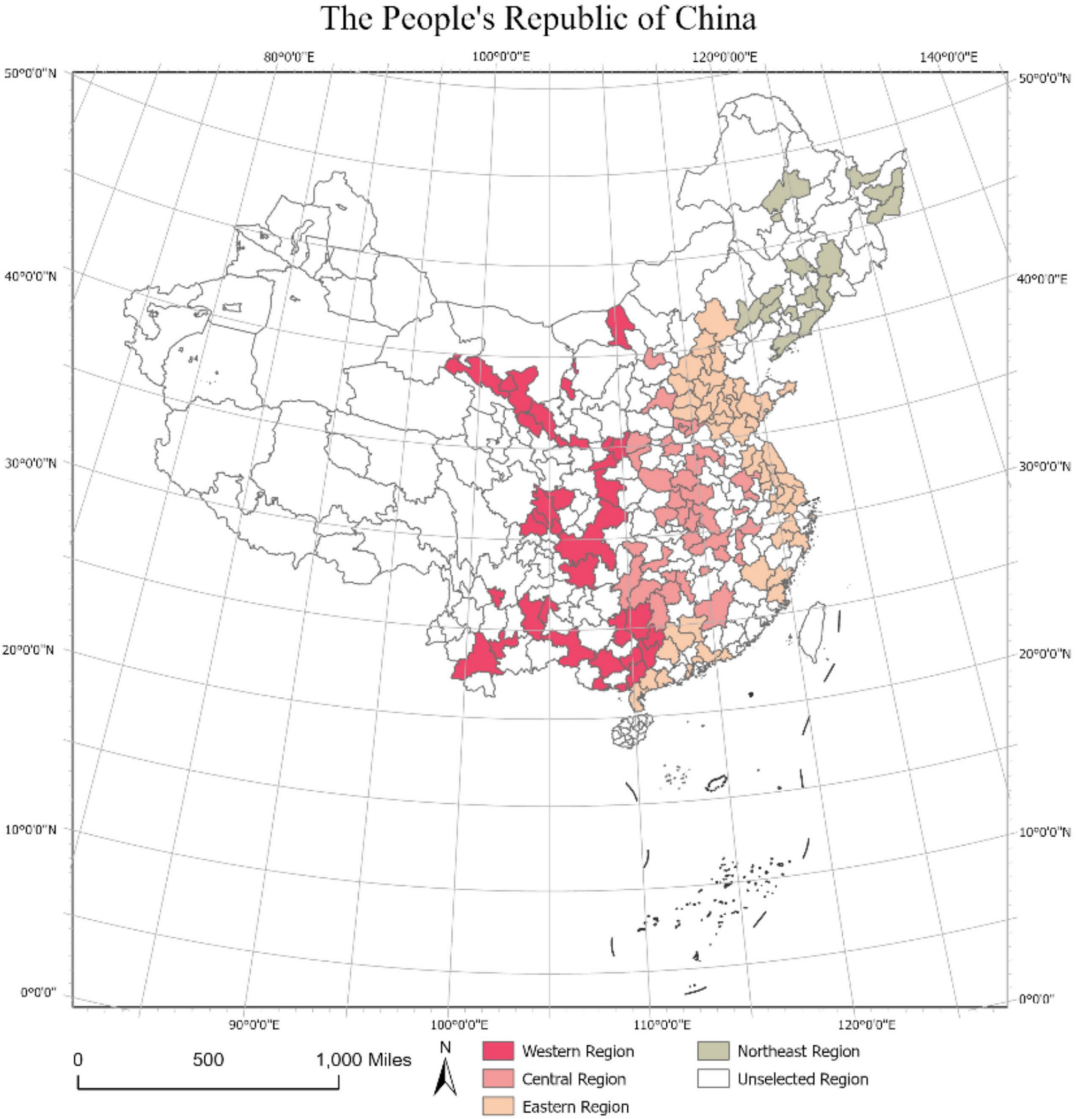
The locations of the four geographic segments and their studied cities in China. The map is created based on the standard map with the map content approval number: No.GS(2019)1822, and the base map is unaltered (48). Data available from: https://cloudcenter.tianditu.gov.cn.

The phenomenon of low fertility rates has attracted considerable attention from researchers in a variety of disciplines. Past research on the factors influencing fertility intention has mainly examined their effects separately at different levels of abstraction (44–46). Such an isolated view cannot reveal the effects of interactions among these factors on people’s fertility intention. Another limitation in current research is that the fertility intention distinction might not be clear owing to regional diversities throughout a country, such as China. Therefore, the factors influencing the fertility intention in different parts of the country must be revisited through a novel lens. Given this, we aim to reconcile the mixed effects of using different sources of data to examine fertility attention in a holistic manner by applying the Poisson regression model. The conceptual framework of this study focuses on the relationship between housing status and fertility intention, as shown in Figure 1. We also hypothesize that these effects on fertility intention vary when taking regional disparity into account. This work represents an initial empirical attempt to aggregate factors influencing fertility intention at the microscopic (i.e., personal attributes, personal health, and housing mortgage), mesoscopic (i.e., social environment), and macroscopic levels (i.e., urban built environment). Linking the housing status to fertility intention can yield suggestions to enhance the overall fertility levels in China and alleviate the burden imposed by the country’s aging population.

## 2 Materials and methods

### 2.1 Study area and data sources

The data for this study were retrieved from the China Labor-force Dynamics Survey (CLDS) 2018, a nationally representative large-scale tracking survey in China targeting the labor force that the Social Science Survey Center of Sun Yat-sen University initiated. The dataset contained tracking and cross-sectional data at three levels: individual laborer, household, and community. It was designed to systematically monitor the social structure of village communities and the changes in interactions among households and individual laborers through a biennial tracking survey of urban and rural villages in China (47). The survey used multi-stage and multi-level probability samples that were proportional to the size of the labor force and focused on the systematic detection of the residential communities, households, education, employment, labor rights, occupational mobility, and occupational protection and health of the labor force. Samples of the CLDS cover almost all areas of China (excluding Hong Kong, Macao, Taiwan, Tibet, Hainan, and Xinjiang), and contain information on 368 communities, 13,501 households, and 16,537 individual members of the labor force.

**Figure 2:**
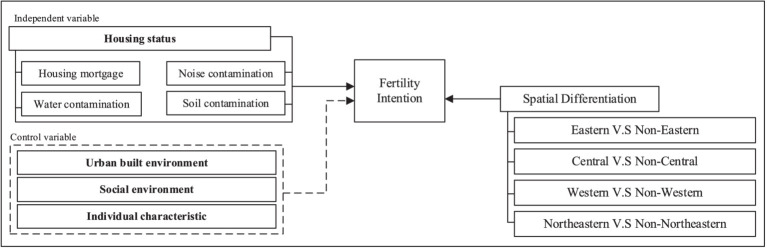
Conceptual framework.

The geographical segmentation of the study area is provided in Table 1. We collected information on the personal attributes, work status, individual health, social interactions, and fertility intention of residents in the four geographic segments from the dataset (Figure 2). After excluding data with missing values, the final sample contained 7,512 items, including 488 from the northeast of China, 3,198 from its east, 1,701 from central China, and 2,125 in the west of the country.

**Table 1:** Geographic segments and their studied cities.

Geographic segments	Places
Western Region	Inner Mongolia Autonomous Region, Guangxi Zhuang Autonomous Region, Chongqing Municipality, Sichuan Province, Guizhou Province, Yunnan Province, Shaanxi Province, Gansu Province, Qinghai Province, Ningxia Hui Autonomous Region
Central Region	Shanxi Province, Anhui Province, Jiangxi Province, Henan Province, Hubei Province, Hunan Province
Eastern Region	Beijing Capital, Tianjin Municipality, Hebei Province, Shanghai Municipality, Jiangsu Province, Zhejiang Province, Fujian Province, Shandong Province, Guangdong Province
Northeast Region	Heilongjiang Province, Jilin Province, Liaoning Province

### 2.2 Variable selection

We selected four dimensions related to the change in people’s fertility intention based on the relevant literature: housing status, urban built environment, social environment, and individual characteristics (Figure 2). These dimensions were directly retrieved from the CLDS 2018 questionnaire.

#### 2.2.1 Dependent variable

The respondents from CLDS2018 were quizzed with a series of questions about the number of children and future fertility intentions, which were asked either under the current fertility policy or in a completely autonomous environment. These metrics, both quantity and willingness, are commonly used to realize fertility rates and trends (48, 49). For this question can be well adapted to reflect the real intention of residents, the fertility intentions of residents in our study are measured using responses to the question ‘How likely are you to have children in the future if the fertility policy were completely liberalizing?’, which is from the CLDS 2018 questionnaire. The research sample for this study thus consisted of data on Chinese adults aged 20 to 45, which focused primarily on the physiology of the respondents according to when the average adult can reproduce. However, the likelihood of having a first child in China is minimal among adults aged 18 and 20 years, while adults over 45 years of age are considered beyond a suitable age for reproduction.

The willingness to have children of all respondents in the areas selected in the case study was classified into five levels: “1” represented “unwillingness to have children,” “2” denoted “willingness to have one child,” “3” represented “willingness to have two children,” “4” represented “willingness to have three children,” and 5 denoted “willingness to have four or more children.” A higher value on the numerical scale implied a stronger fertility intention among the residents. This indicator is considered a continuous variable in the later statistical analysis model.

#### 2.2.2 Independent variables

The social environment is significantly related to people’s fertility intention. We used the perception of well-being (Life well-being, life satisfaction, and economic satisfaction) and the environment of the community (sense of safety) to reflect the social environment of the respondents (Table 2). The perception of community safety consisted of a question about specific aspects of community safety and was designed to understand how worried residents were about the occurrence of safety-related incidents in their communities. The overall score on the answers in Table 2 ranged from 1 to 5, with higher scores indicating higher safety and well-being. It was a five-point scale consisting of the items “never,” “rarely,” “sometimes,” “more often,” and “often.” The same indicator has been similarly employed in prior research (48, 49).

**Table 2:** Measured variables.

Measured variables	Items
Housing status	
Housing mortgage	Do you have any debts related to housing?
Water contamination	How bad do you think the water pollution is where you live?
Noise contamination	How bad do you think the noise pollution is where you live?
Soil contamination	How bad do you think the soil pollution is where you live?
Social Environment	
Community safety	What do you think is the overall level of social security?
Life well-being	In general, do you think your life was happy?
Life satisfaction	In general, are you satisfied with your life?
Economic satisfaction	In general, are you satisfied with your income?

People’s fertility intention are also associated with their health status, especially in the case of families without children. We thus considered factors related to people’s mental health. We accordingly designed a questionnaire consisting of 20 questions on symptoms that might have occurred in the week prior to filling it out. All 20 items on the questionnaire were negative indicators, and the participants’ responses were scored on a four-point scale: “not/mostly not (less than 1 day),” “rarely (1 to 2 days),” “often (3 to 4 days),” and “almost always (5 to 7 days).” The overall score on the 99 questionnaire ranged from 20 to 80. The lower the score on the scale was, the higher the level of mental health of the relevant respondent. Values of Cronbach’s alpha of the mental health and community safety were 0.942 and 0.851, respectively. The values of these coefficients were smaller than those of the original coefficients following the removal of certain items, indicating that all three scales were highly reliable (Table 3).

**Table 3:** Measured variables of mental health and community safety.

Constructed dimensions	Cronbach’s alpha	Standardized Cronbach’s alpha	Description
Mental health	0.942	0.943	Get upset over little things
			Loss of appetite
			Get help still cannot get rid of the depressed mood
			Not as good as most people
			Cannot concentrate on things
			Feel down
			It feels like everything is so hard
			Feel hopeless about the future
			Feel life is a failure
			Feel scared
			Poor sleep
			Feeling unhappy
			Talk less than usual
			Feel lonely
			Feel people are unfriendly
			Feel life is not interesting
			Used to cry
			Feel sad
			Feel People do not like me
			Feel life cannot go on
Community safety	0.851	0.857	Feel hanging around outside is not safe
			Feel going out alone at night is not safe
			Failure to lock doors and Windows is a risk of burglary
			Exposing money risks being targeted
			Children who go out alone are at risk of being trafficked

The core explanatory variables focused on the housing status of the respondents, including the housing mortgage and the objective residential environment (Table 2). The housing mortgage was used to reflect the affordability of housing for residents in each city. We considered the average sale price of commercial houses sold in cities above the prefecture level from the macroeconomic and real estate database of the National Information Center as the average housing price. The objective residential environment is related to the residents’ fertility intention by controlling their health. We thus considered the degree of pollution of the living environment as another core explanatory variable in the model.

Furthermore, indicators of the urban built environment from the Yearbook of China City Statistical in 2016 are considered in this study, including urbanization rate, net primary productivity (NPP), and population density. NPP is frequently employed as a quantitative indicator of vegetation growth (50, 51). This indicator was used in the model to evaluate the quality of green space in the selected area. The indicators mentioned above have been frequently cited in previous research relevant to fertility intention, but their direct relationship with intention has not been revealed. The control variables used in this paper were indicators of personal attributes. Demographic characteristics (gender, age, marital status, political status, maternity insurance, education, and number of migratory movements) were used in the model as control variables (Table 4).

**Table 4:** Sociodemographic characteristics of the entire sample and the samples from eastern, central, western and northeastern regions.

Variables	Directions	Total		East		Mid		West		North East	
		Fre.	Pro.	Fre.	Pro.	Fre.	Pro.	Fre.	Pro.	Fre.	Pro.
Gender	Male	3,306	44.0%	1,342	42.0%	731	43.0%	1,025	48.2%	208	42.6%
	Female	4,206	56.0%	1856	58.0%	970	57.0%	1,100	51.8%	280	57.4%
Marital status	Single	5,656	75.3%	2,369	74.1%	1,326	78.0%	1,636	77.0%	325	66.6%
	Non-single	1856	24.7%	829	25.9%	375	22.0%	489	23.0%	163	33.4%
Political status	MCPC	444	5.9%	212	6.6%	83	4.9%	121	5.7%	28	5.7%
	Non-MCPC	7,068	94.1%	2,986	93.4%	1,618	95.1%	2004	94.3%	460	94.3%
Maternity insurance	Purchase	1,133	15.1%	647	20.2%	245	14.4%	187	8.8%	54	11.1%
	Not purchase	6,379	84.9%	2,551	79.8%	1,456	85.6%	1938	91.2%	434	88.9%
Education	No education/primary school	1978	26.3%	714	22.3%	383	22.5%	763	35.9%	118	24.2%
	Junior/senior high school	3,726	49.6%	1,590	49.7%	890	52.3%	982	46.2%	264	54.1%
	Undergraduate college	1,653	22.0%	814	25.5%	403	23.7%	347	16.3%	89	18.2%
	Master/Doctor	112	1.5%	50	1.6%	18	1.1%	27	1.3%	17	3.5%

#### 2.2.3 Research model

We constructed a robust Poisson regression model to verify the effects of housing prices on people’s fertility intention, and used Stata 16 software to analyze the results (52, 53). The distribution of error was specified as a Poisson distribution, and the variance in the results was obtained by applying the “sandwich” method. The model could directly analyze on the original data without needing to manipulate it. Robust Poisson regression can be considered when the log-binomial distribution does not converge. Let $y_{i}$ and $x_{i}={\left(x_{i}^{1},x_{i}^{2},\cdots ,x_{i}^{P}\right)}^{T}$ be the i−th $\left(i=1,2,\cdots ,n\right)$ observed dichotomous ending variable and the P × 1-dimensional vector of explanatory variables, respectively. The relationship can then be represented by the Poisson regression model as follows:


$$\log\left(p^{i}\right)=\beta_{0}+\sum_{p=1}^{P}\beta_{p}x_{i}^{p}$$

where $p^{i}=\mathrm{Pr}\left(y_{i}=1/x_{i}\right)\text{.}$ The regression coefficient $\beta_{p}$ represents the corresponding change in $\log\left(p\right)$ for each unit change in the p−th independent variable $x_{p}$ when the other independent variables are controlled.

## 3 Results

### 3.1 Descriptive analysis of housing status and fertility intention

**Figure 3:**
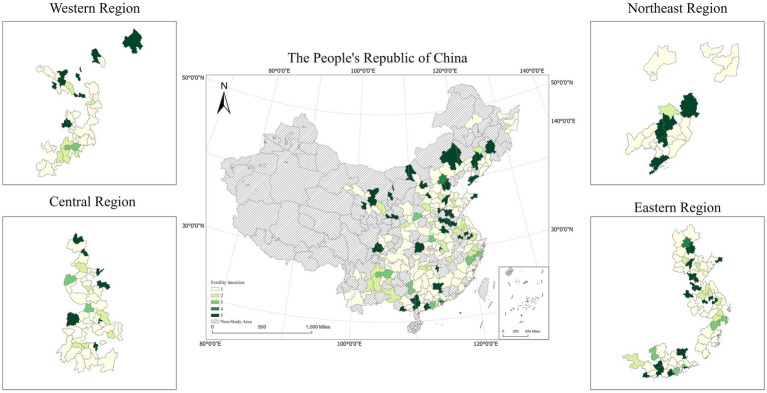
Spatial distribution of average inhabitant’s fertility intention in Chin. The map is created based on the standard map with figure number GS (2019)1822, and the base map is unaltered.

Tables 4, 5 list the social demographic characteristics of the respondents, which also shows the social demographic characteristics of samples from the four geographic segments (western, northeastern, central, and eastern regions of China). In Table 4, the total ratio of males to females was 0.78 to 1, of which 24.70% were married. As shown in Table 5, while the average age of respondents was 34.24 years (S.D. = 7.486), the results in four different regions show similarity to one another. The average number of willing to give birth to children was 1.95 (S.D. = 1.538), which indicates that the majority of respondents are more likely to have no children or no more than one or two children in the future. This suggests a very low level of actual fertility intention in the total sample (Figure 3).

**Table 5:** Mean value of sociodemographic characteristics in the entire sample and the samples from eastern, central, western and northeastern regions.

Variables	Total		East		Mid		West		North East	
	Mean	S.D.	Mean	S.D.	Mean	S.D.	Mean	S.D.	Mean	S.D.
Age	34.24	7.486	34.60	7.136	34.34	7.630	33.56	7.926	34.49	7.050
Community security	1.76	0.596	1.79	0.612	1.82	0.548	1.68	0.598	1.71	0.617
Life well-being	3.80	0.899	3.84	0.912	3.71	0.914	3.74	0.874	4.03	0.806
Life satisfaction	3.67	0.900	3.71	0.923	3.64	0.861	3.62	0.872	3.80	0.979
Economic satisfaction	3.21	1.016	3.29	1.006	3.21	0.948	3.04	1.030	3.50	1.119
Desire to have children	1.95	1.538	2.03	1.581	1.92	1.583	1.87	1.436	1.84	1.504
Mental health	27.20	8.457	26.64	8.189	27.29	8.507	28.16	8.857	26.41	7.836
Water contamination	2.93	0.830	2.86	0.788	2.88	0.820	3.10	0.881	2.77	0.803
Noise contamination	3.07	0.807	3.00	0.772	2.99	0.805	3.26	0.820	2.98	0.840
Soil contamination	3.14	0.729	3.10	0.677	3.11	0.715	3.27	0.803	3.05	0.709
Migratory movements	0.25	0.506	0.25	0.519	0.25	0.477	0.26	0.532	0.14	0.384

There were large differences in the level of education of residents of different regions. Compared with the overall sample, residents of the eastern region had a higher level of education while those of the western region had a lower level of schooling. In the eastern region, 27.01% of the residents had been educated to a higher level than the national average. A majority of residents (82.11%) in the western region had completed secondary education and lower. The difference between the regions in terms of marital status was not prominent, with 73.01% of the residents in the northeast region having been married, which was slightly lower than the values for the other three regions. Table 5 shows that the mean intensity of fertility intention of the total sample was 1.95. Among the sub-samples, the northeast region had the lowest mean value of 1.84. This was owing to the relatively high education and rate of employment of women in this region as well as the more thorough implementation of family planning policies in the past, which weakened their fertility intention. The remaining three regions had similar mean values of fertility intention: 1.87 in the western region, 1.92 in the central region, and 2.03 in the eastern region.

### 3.2 Effects of housing status on fertility intention

Table 6 shows that the rate of urbanization, NPP, housing mortgage, life satisfaction, mental health, and degree of water contamination were significantly correlated with the residents’ fertility intention. An increase in urbanization rate (Coeff. = 0.149, *p* = 0.008<0.05) and NPP (Coeff. = 0.148, P < 0.001) causes a similar rise in fertility intention. The positive effects of green space on subjective well-being, sense of community safety, community satisfaction, and place attachment, which contribute to residents’ fertility intention, have been documented in numerous studies, including our study (43, 54–58). People who did not have a housing mortgage had higher fertility intention than those who did. The presence of a housing mortgage reduced their fertility intention by 0.05 points (*p* = 0.001<0.05). This implies that reproductive intention is not only related to property ownership, but also associated with the presence of a housing mortgage (59, 60). Even though the results of this study find that higher urbanization rates are accompanied by an increase in fertility intention, in China, high urbanization also means high house prices (61–63). People in China prefer homeownership to renting, which is closely associated with the pursuit of stability in life, and can be attributed to the deeply ingrained traditional Chinese notion of the family (64). Hence, despite the swift escalation in prices in the housing market, young individuals prefer acquiring personal property even if this necessitates a substantial housing loan (26). Properties in desirable locations in certain metropolitan areas frequently command prices significantly above the prevailing market rates due to their association with high-quality children’s education (65). This inevitably contributes to higher living expenses for families aspiring to provide their children with quality education, consequently reducing their inclination to have additional offspring. Numerous investigations have substantiated this claim (48). However, studies indicate that higher housing prices can increase fertility intention among mortgage holders (59).

**Table 6:** Results of regression analysis of factors influencing fertility intention of the entire sample.

Variables	Coeff	Robust S.E	Z
Housing status			
Housing mortgage	−0.048**	(0.014)	−3.29
Water contamination	−0.028**	(0.010)	−2.71
Noise contamination	−0.011	(0.011)	−0.98
Soil contamination	0.002	(0.013)	0.17
Urban built environment			
Urbanization rate	0.149**	(0.056)	2.65
Net primary productivity	0.148***	(0.038)	3.87
Population density	−0.000	(0.000)	−0.31
Social Environment			
Community safety	0.003	(0.010)	0.17
Life well-being	0.006	(0.010)	0.32
Life satisfaction	0.021^+^	(0.011)	1.94
Economic satisfaction	0.010	(0.008)	1.25
Individual Characteristic			
Mental health	0.003***	(0.001)	3.75
Age	−0.028***	(0.001)	−22.44
Gender	−0.100***	(0.015)	−6.71
Marital status	−0.585***	(0.022)	−26.89
Political status	−0.093**	(0.035)	−2.68
Migratory movements	−0.014	(0.016)	−0.89
Junior or senior high school	0.128***	(0.018)	6.98
Undergraduate college	0.168***	(0.026)	6.52
Master or Doctor	0.255***	(0.054)	4.73
Maternity insurance	0.014	(0.022)	0.62
_cons	1.800***	(0.092)	19.51
N	7,512		

Standard errors in parentheses ^+^*p* < 0.1, *p < 0.05, **p < 0.01,***p < 0.001; Robust S. E is the robust standard error of regression coefficients.

Higher levels of life satisfaction (*p* = 0.052<0.1) and mental health (*p* < 0.01) were positively associated with a strong fertility intention. Specifically, a one-point increase in life satisfaction and mental health yielded deficits of 0.019 points and 0.003 points, respectively, in fertility intention. Moreover, the greater the level of water pollution in the surroundings of a given residential area was, the lower the fertility intention of the population, with each unit increase in the level of pollution accompanied by a 0.02 unit decrease in their fertility intention. Another urban built environment indicator, NPP, is positively correlated to fertility intention.

A higher age (*p* < 0.001) and lower education (*p* < 0.001) were also associated with lower fertility intention. The overall fertility intention among women was lower than among men and was much higher in married people than in single people. These may be due to factors such as level of education, work circumstances, and poor performance in raising children with the spouse (13, 29, 60, 66). Marital life satisfaction, good communication with husbands regarding childbearing, and family income are associated with women’s willingness to have a first child. In contrast, the work situation inhibits women’s willingness to have a second child. The absence of a job and gender inequality in the division of housework inhibit women’s willingness to have a third child (67). However, some studies have found that the effect of differences in fertility intention between men and women is primarily caused by house prices, with men exhibiting lower fertility intention in areas with higher house prices and women exhibiting the opposite trend (22). Although our study found that overall fertility intention also increased with higher levels of education, this may suggest that higher levels of education are associated with higher personal incomes and, consequently, have a reduced inhibiting effect on fertility intention. However, research conducted in the Nordic region has revealed that the higher a woman’s level of education, the lower her fertility intention (68). Also, the rising cost of housing, and governmental policies on fertility is a daunting obstacle to couples’ willingness to have children. Respondents who were the members of the Communist Party exhibited lower fertility intention than non-party members, where this may be related to the planned parenthood policy implemented from 1979 to 2015 (3, 69).

In addition, the availability of maternity insurance did not exhibit a direct significant relationship with fertility intention (*p* = 0.537 > 0.1). Only 15.1% of the cohort had maternity insurance coverage in the total sample (70, 71). According to a study conducted in China, increased public financial support effectively increased fertility intention (72). As a result, the findings of this study may show that maternity insurance has no natural effect on fertility intention in practice. Overall, the number of community household transfers did not show a significant effect, but it has been suggested that mobility affects fertility intention (73–76). Whether there are differences between regions will be further examined in subsequent sections.

### 3.3 Housing status and fertility intention: regional differences

We used Fisher’s permutation test based on the bootstrap test to identify the mechanism of divergence in the effects of fertility intention between regions. The results in Table 7 show considerable regional disparities in the affordability of housing as well as the urban built environment and the objective environments of the neighborhood. Fertility intention in the east of China were linked to the urbanization rate, housing mortgage, subjective well-being, life satisfaction, and water contamination. Those in its northeastern region were related to soil contamination and mental health, while fertility intention was highly associated with all the socioeconomic environment factors in the central region.

**Table 7:** Differential analysis results of samples from eastern, central, western and northeastern regions.

	Non-East (*N* = 4,314)		East (*N* = 3,198)		SCD	Non-Mid (*N* = 5,811)		Mid (*N* = 1701)		SCD	Non-West (*N* = 5,387)		West (*N* = 2,125)	SCD		Non-NEast (*N* = 7,024)		NEast (*N* = 488)		SCD
	Coef.	S.E.	Coef.	S.E.		Coef.	S.E.	Coef.	S.E.		Coef.	S.E.	Coef.	S.E.		Coef.	S.E.	Coef.	S.E.	
Housing status																				
housing mortgage	−0.108^**^	−3.02	−0.105^*^	−2.39	0.477	−0.084^**^	−2.61	−0.145^**^	−2.64	0.189	−0.164^***^	−4.90	0.0815^+^	−1.67	0.000	−0.092^**^	−3.18	−0.162	−1.33	0.287
Water pollution	0.028	−1.02	−0.121^***^	−3.73	0.000	−0.068^**^	−2.86	−0.001	−0.02	0.07	−0.073^**^	−2.93	−0.023	−0.59	0.134	−0.070^**^	−3.27	0.031	−0.35	0.11
Noise pollution	0.033	−1.20	−0.035	−1.07	0.09	0.043^+^	−1.79	−0.087^*^	−2.10	0.002	−0.035	−1.42	0.135^***^	−3.34	0.000	−0.002	−0.11	0.064	−0.78	0.238
Soil contamination	−0.069^*^	−2.18	0.063^+^	−1.62	0.003	−0.032	−1.14	−0.04	−0.74	0.451	0.041	−1.34	−0.042	−0.96	0.05	0.007	−0.28	−0.240^*^	−2.43	0.009
Urban built environment																				
Urbanization rate	−0.002	−0.02	0.338^+^	−1.73	0.065	0.349^**^	−2.97	−0.163	−0.50	0.022	0.407^**^	−2.92	−0.003	−0.02	0.044	0.202^+^	−1.81	0.589	−1.09	0.218
Net primary productivity	0.299^**^	−2.8	0.033	−0.24	0.030	0.312^***^	−3.63	0.379	−1.32	0.351	0.127	−1.14	0.209^+^	−1.67	0.321	0.211^*^	−2.55	0.39	−0.5	0.28
Population density	−0.001	−1.63	0.000	−0.9	0.043	0.000	−0.96	−0.002^***^	−3.60	0.000	0.000	−0.37	−0.001	−1.05	0.218	0.000	−1.29	0.000	−0.36	0.221
Socioeconomic factors																				
Community security	0.009	−0.28	0.027	−0.73	0.327	0.055^*^	−2.08	−0.179^***^	−3.49	0.000	0.005	−0.18	0.091^*^	−2.17	0.056	0.023	−0.92	0.058	−0.58	0.344
Life well-being	−0.115^***^	−4.17	0.219^***^	−6.7	0.000	0.108^***^	−4.52	−0.205^***^	−4.52	0.000	0.071^**^	−2.79	−0.069^+^	−1.81	0.003	0.032	−1.47	−0.002	−0.02	0.35
Life satisfaction	0.122^***^	−4.11	−0.113^**^	−3.19	0.000	−0.028	−1.12	0.148^***^	−2.96	0.000	−0.019	−0.68	0.010^***^	−2.53	0.008	0.027	−1.17	0.028	−0.3	0.493
Economic satisfaction	0.011	−0.54	0.035	−1.33	0.224	0.009	−0.49	0.138^***^	−3.95	0.000	0.032	−1.59	−0.01	−0.36	0.109	0.037^**^	−2.15	−0.074	−1.11	0.029
Individual level factors																				
Mental health	0.010^***^	−4.82	0.002	−0.69	0.002	0.008^***^	−3.99	0.002	−0.5	0.072	0.004^+^	−2.06	0.011^***^	−3.6	0.05	0.005^**^	−3.09	0.015^+^	−1.84	0.075
Age	−0.048^***^	−17.89	−0.061^***^	−14.98	0.002	−0.053^***^	−20.27	−0.047^***^	−10.63	0.148	−0.061^***^	−21.37	−0.041^***^	−10.85	0.000	−0.051^***^	−21.93	−0.052^***^	−5.99	0.498
Gender	−0.178^***^	−4.79	−0.197^***^	−4.40	0.372	−0.156^***^	−4.74	−0.170^**^	−2.95	0.397	−0.231^***^	−6.70	−0.062	−1.16	0.006	−0.150^***^	−5.02	−0.271^*^	−2.36	0.154
Marital status	−1.636^***^	−32.36	−1.250^***^	−19.81	0.000	−1.251^***^	−27.90	−2.232^***^	−26.81	0.000	−1.475^***^	−31.49	−1.399^***^	−18.25	0.25	−1.553^***^	−37.07	−0.920^***^	−6.98	0.001
Political status	−0.186^**^	−2.16	−0.372^***^	−3.92	0.082	−0.351^***^	−4.92	0.019	−0.14	0.012	−0.292^***^	−3.81	−0.266^+^	−2.26	0.435	−0.195^**^	−2.97	−0.762^**^	−2.71	0.02
Migratory movements	0.049	−1.38	−0.117^**^	−2.60	0.000	−0.080^**^	−2.49	0.103^+^	−1.77	0.005	−0.017	−0.49	−0.051	−1.06	0.299	−0.067^*^	−2.36	0.366^**^	−2.61	0.000
Junior or senior high school	0.108^*^	−2.56	0.369^***^	−6.26	0.000	0.296^***^	−7.52	−0.048	−0.71	0.000	0.197^***^	−4.59	0.256^***^	−4.24	0.156	0.209^***^	−5.83	0.217^+^	−1.67	0.458
Undergraduate college	0.098	−1.56	0.771^***^	−9.52	0.000	0.655^***^	−11.44	−0.281^**^	−2.85	0.000	0.408^***^	−6.79	0.241^**^	−2.61	0.096	0.316^***^	−6.14	1.218^***^	−6.05	0.000
Master or Doctor	0.522^**^	−3.21	0.848^***^	−4.39	0.128	0.859^***^	−6.19	0.259	−0.93	0.041	0.416^**^	−2.84	1.086^***^	−4.52	0.012	0.676^***^	−5.06	0.894^+^	−2.24	0.362
Maternity insurance	0.07	−1.10	−0.237^***^	−3.75	0.001	−0.036	−0.71	−0.082	−0.94	0.345	−0.185^***^	−3.68	0.511^***^	−5.05	0.000	−0.058	−1.27	−0.24	−1.23	0.183
_cons	4.781^***^	−20.67	4.579^***^	−13.81	0.294	3.957^***^	−18.42	6.398^***^	−16.58	0.000	5.085^***^	−21.64	3.305^***^	−10.38	0.000	4.611^***^	−23.86	4.150^***^	−4.68	0.254

Standard errors in parentheses. ^**+**^*p* < 0.1, *p < 0.05, **p < 0.01, ***p < 0.001; NEast means Northeast region of China, SCD means Significance of component differences. S.E is the standard error of regression coefficients using bootstrap test.

Specifically, there was a more significant difference between the eastern and central regions, and the western region in terms of the relationship between mortgage loans and fertility intentions (p was significant at the 5% level). Fertility intention in the western region was positively affected by the mortgage, but those in the east and central regions were significantly negatively affected by it. A significant difference was noted in the effects of community safety between the central and western regions, and the eastern and northeastern regions. The central region exhibited a negative relationship between community safety and fertility intention, the west had a positive relationship, and the east and northeast exhibited no significant relationship between community safety and the residents’ fertility intention. The correlation between life satisfaction and fertility intention was positive among residents in the eastern, central, and western subregions but not among those in the northeastern region. Economic satisfaction was positively related to fertility intention only in the central region, while they were positively associated with the residents’ life satisfaction in the western region and central region but not in the eastern region. The decline in the willingness to have children among residents in the eastern region was primarily attributable to the increase in water contamination, while that in the central region was attributable to an increase in noise pollution. In addition, a modest positive correlation (significant at the level of 0.1) was noted between soil contamination and the fertility intention of residents in the east areas, while soil contamination was negatively correlated with fertility intention in the northeast. The northeastern region has more crude industries, which constitute a significant source of soil contamination, and residents whose living environments were more severe had lower fertility intention. This was most likely because long-term soil contamination in residential areas can negatively impact the health of future generations.

There are also regional differences in the built environment. The difference in the effect of urbanization rate is mainly between the eastern and non-eastern regions (SCD = 0.065 < 0.1). There is a significant positive correlation between the urbanization rate and the fertility intention of the eastern residents (the coefficient is 0.338), and the higher the urbanization rate, the higher the fertility intention, which may be related to the fact that the residents of the eastern region have a higher income and economic level than the other regions. The contributions of NPP to fertility intention mainly appear in the western region, with a significant positive effect, while the other regions show a non-significant difference (SCD = 0.321 > 0.1). For every point increase in NPP, the fertility intention of western residents will increase by 0.209 percentage points. This pattern is consistent with existing studies that NPP in China shows a decreasing trend from southeast to northwest in spatial distribution (77, 78). These suggest that improving the residential green environment in the western region is more conducive to increasing the fertility intention of residents. However, there is no relationship shown in the eastern region (SCD = 0.03 < 0.05). Although the increase in population density did not affect fertility intention in the total sample, it showed an inhibitory effect in the central region (the coefficient is −0.002), but not in any of the other three regions (SCD < 0.01).

## 4 Discussion

Understanding how inhabitants’ fertility intention is affected by urban development factors is critical to informing policies and interventions after a continuing decline in the birth rate. In recent years, the notion of ‘expectant but fear for bear’ has increasingly become prevalent among childbearing couples. Due to a variety of economic constraints, residents may be incapable of bearing the numerous expenses associated with childbirth. Housing expenses, a substantial proportion of the daily expenditures of residents, have a strong correlation between housing conditions and housing costs, which is profoundly related to the inclination of residents’ fertility. From a housing function perspective, subsidized housing features low-cost and small apartments that might not provide suitable conditions for raising two or more children. By comparison, those who are suitable for families bearing several children also require an increased housing cost or being positioned farther away from downtown. Furthermore, while raising a child, the demand for a higher quality education close to home might considered as well. Those who provide well-education services and infrastructures in communities also meet higher prices, which in turn increases the financial burden on bearing. Naturally, individuals living in a particular area where they could make a balance between well-bearing, housing conditions, and living expenses might come at the cost of economic opportunities. These phenomena on the fertility intention of Chinese families have been widely discussed in the research. We provided empirical evidence to explain the low fertility intention among people in China by examining the effects of the booming real housing market and housing mortgages.

Previous studies have generally evaluated the correlation between increasing housing prices and inhabitants’ fertility intention, with housing prices as an indicator of the average cost of residential units in neighborhoods or cities. Due to the varying role of house property ownership in fertility intention, housing prices are less powerful than housing loans in reflecting the actual housing costs undertaken by residents. Also, a few studies have addressed aggregated factors at the individual level (including personal attributes, housing mortgage, personal health, and social environment) and the municipal level (i.e., urban built-up environment). Hence, this study used the existence of a housing loan, other housing status, built environment, social environment, and personal attributes as the measures. The findings in this study contribute to an in-depth understanding of the correlation mechanism on fertility intention in China by revealing different dimensions of residents’ living environments, especially the housing dimension, which includes both spatial and economic factors.

The findings of this study also provide recommendations for the government to enhance residents’ fertility intention. Our experimental results that showing the mechanism of lower fertility intention in China may help the government ameliorate incentives for childbearing to enhance residents’ willingness to have more children. Housing policies should be adjusted based on the demographic structure and geographic divergence of the country. While strengthening its efforts for market supervision, it is also necessary for the government to reduce the difficulty of homeownership for families with housing needs. Some measures can be taken, such as reducing the restrictions on residents’ purchase of houses, adjusting tax policies, lowering the interest rate of bank housing loans, and perfecting the housing security system. Also, the higher-pricing houses in good catchment communities brought about by the inequity in children’s education should be solved by improving the compulsory education system and relevant childcare infrastructure for children in different types of communities. The inequalities in genders can be less pronounced by extending the length of maternity leave for women from 98 days to 150 or 180 days. More importantly, increasing paid paternity and maternity leave from work benefits should be extended equally to couples, which is encouraged to reduce bearing costs and insidious job prejudice for women (79, 80).

## 5 Conclusion

This study analyzed the relationship between fertility intention and housing status in China, employing a cross-sectional dataset from the China Labor Dynamics Survey of 7,512 respondents. The primary aim was to investigate the associated factors of the fertility intention among residents, specifically focusing on the effect of housing and environmental variables. The second purpose was to compare the groups in various geographical regions, particularly concerning the differential mechanisms of relevant intention intensity. Our empirical findings, obtained using a Poisson regression model and Fisher’s permutation test, address these objectives and suggest the following. (1) In general, the results in this study find that urbanization rate, NPP, life satisfaction, mental health, and education are positively associated with people’s fertility intention in the overall sample, while the housing mortgage, age, and water contamination negatively affected them. (2) The results indicate a significant gender difference in fertility intention, with women having lower fertility intention than men. (3) We also highlighted the differences among the four geographic segments exhibiting regional diversity. Overall, there are no significant regional differences in housing status, and it is worth noting that residents with mortgages show higher fertility intention in the western region. (4) In terms of pollution in the residential environment, residents in the eastern part of the country are mainly affected by water contamination. In contrast, residents in the northeastern part of the country are mainly affected by soil contamination. Although the urbanization rate does not significantly affect fertility intention in the western regions, the noise pollution and mortgage indicators point to the possibility of such a relationship, as urbanized areas are generally more polluted and have higher housing prices than suburban areas. However, this inference needs to be further confirmed in the future. (5) Concerning the urban built environment, the effect of NPP on fertility intention is insignificant in the East. (6) In particular, we find a lower fertility intention among residents with higher life satisfaction in the East, which is the opposite of the other regions. At the same time, fertility intention is lower for residents with maternity insurance in the eastern region, which is the opposite of the western region, further confirming the low utility of maternity insurance in practice.

Overall, this study thoroughly examines the associated factors of housing conditions and family fertility, considering individual economic factors, social environment, and urban environment, as well as regional differences of associated factors of residents’ fertility intentions. There are still limitations. It is important to note that the data used in this study are cross-sectional. This may unavoidably lead to random errors or measurement errors in analysis, though their impact on the accuracy of the final results is relatively minor. Additionally, our study is unable to determine causality between the urban built environment and changes in residents’ fertility intentions from a longitudinal perspective.

## Data Availability

The raw data supporting the conclusions of this article will be made available by the authors, without undue reservation.
