# Corrigendum: Does inhabitant's fertility intention respond to housing status in the urban built environment: evidence from China

**DOI:** 10.3389/fpubh.2025.1608694

**Published:** 2025-04-29

**Authors:** Xuefang Zhuang, Qingyin Li, Tian Su, Siying Zhang, Rong Wu, Tianxiang Zheng

**Affiliations:** ^1^School of Architecture and Urban Planning, Guangdong University of Technology, Guangzhou, China; ^2^Shenzhen Tourism College, Jinan University, Shenzhen, China; ^3^Department of E-Commerce, Jinan University, Shenzhen, China

**Keywords:** fertility intention, housing status, housing mortgage, regional diversity, mental health

In the published article, there was an error in affiliations 1, 2, 3.

For affiliation 1: Instead of “Yuexiu Branch of Guangzhou Urban Planning & Design Survey Research Institute, Guangzhou, China”, it should be “School of Architecture and Urban Planning, Guangdong University of Technology, Guangzhou, China”.

For affiliation 2: Instead of “School of Architecture and Urban Planning, Guangdong University of Technology, Guangzhou, China”, it should be “Shenzhen Tourism College, Jinan University, Shenzhen, China”.

For affiliation 3: Instead of “Shenzhen Tourism College, Shenzhen Campus, Jinan University, Shenzhen, China”, it should be “Department of E-Commerce, Jinan University, Shenzhen, China”.

In the published article, there was an error regarding the numbered superscripts for all authors. The affiliation of Xuefang Zhuang, Qingyin Li and Rong Wu should belong to 1 School of Architecture and Urban Planning, Guangdong University of Technology, Guangzhou, China. The affiliation of Tian Su and Siying Zhang should belong to 2 Shenzhen Tourism College, Jinan University, Shenzhen, China. The affiliation of Tianxiang Zheng should belong to 3 Department of E-Commerce, Jinan University, Shenzhen, China.

The authors apologize for this error and state that this does not change the scientific conclusions of the article in any way. The original article has been updated.

